# Intimal flap detected by three-dimensional curved multiplanar reconstruction image in isolated posterior inferior cerebellar artery dissection: a report of two cases

**DOI:** 10.1186/s12883-019-1309-3

**Published:** 2019-04-27

**Authors:** Yong Soo Cho, Pahn Kyu Choi, Hyun Ju Seon, Dong Hun Kim, Byoung-Soo Shin, Hyun Goo Kang

**Affiliations:** 1 Department of Radiology, Chosun University School of Medicine, Gwangju, South Korea; 2 Department of Neurology, Chosun University School of Medicine, Gwangju, South Korea; 3 Department of Neurology and Research Institute of Clinical Medicine of Chonbuk National University - Biomedical Research Institute of Chonbuk National University Hospital, Jeonju, South Korea

**Keywords:** Arterial dissection, High-resolution vessel wall MRI, Isolated PICA dissection, Multiplanar reconstruction

## Abstract

**Background:**

Spontaneous isolated posterior inferior cerebellar artery (PICA) dissection has been reported more frequently since high-resolution vessel wall magnetic resonance imaging (HR vw-MRI) was introduced to the field. The intimal flap or double lumen, which is commonly reported to be a direct sign of the dissection, is not easily detectable on HR vw-MRI because the size of the PICA is very small and tortuous.

**Case presentation:**

Two patients with posterior circulation ischemic stroke due to spontaneous isolated PICA dissection underwent HR vw-MRI. The curved multiplanar reconstruction image reconstructed using three-dimensional (3D) HR vw-MRI (3D curved MPR imaging) is helpful to observe tortuous blood vessels such as the PICA because it can visualize the entire vessel course in a single plane. In this report, routine HR vw-MRI revealed only an intramural hematoma in both patients. However, 3D curved MPR imaging discovered the intimal flap which was not observed on the routine HR vw-MRI. Therefore, these two patients were diagnosed with spontaneous isolated PICA dissection due to the intimal flap that was observed on the 3D curved MPR image.

**Conclusion:**

HR vw-MRI is useful for the early diagnosis of isolated PICA dissection. Furthermore, we believe that 3D curved MPR imaging could improve the possibility of diagnosing the dissection early because it can easily confirm direct signs such as an intimal flap or double lumen.

## Background

The incidence of spontaneous isolated posterior inferior cerebellar artery (PICA) dissection is very rare. However, it is important to diagnose PICA dissection accurately and as early as possible because it can cause ischemic stroke or subarachnoid hemorrhage. Owing to the recent development of imaging technology, more studies have reported isolated PICA dissection. However, it is still difficult to accurately diagnose PICA dissection because the size of the PICA is very small. Recent studies have diagnosed isolated PICA dissection in patients by using high-resolution vessel wall MRI (HR vw-MRI), in which a T1 hyperintense intramural hematoma was the main finding [1–3]. Although an intramural hematoma is a pathognomonic sign of PICA dissection, it may be necessary to distinguish it from intraplaque hemorrhage. Therefore, it may be important to visualize a direct sign such as an intimal flap or double lumen. This study presents two cases in which an intimal flap was detected on the curved multiplanar reconstruction image reconstructed from three-dimensional (3D) HR vw-MRI (3D curved MPR image).

## Case presentation

### Case 1

A 56-year-old male patient came to the emergency room due to a right-sided occipital headache and vertigo, which suddenly began three days before the visit, and a symptom of tilting to the right, which began one day before the visit. There was no history of an external injury within the past one week. The patient did not have any underlying diseases, history of migraines, or risk factors for stroke, except for smoking. He also did not have a history of taking antiplatelet agents. When the patient visited the emergency room, his blood pressure was 160/100 mmHg, the pulse rate was 62/min, breathing rate was 19/min, and body temperature was 36.5 °C. The results of a neurological examination showed lateropulsion to the right side, hypoesthesia on the right side of the face, ataxia in the right upper and lower limbs, and the NIH stroke scale was 2. The results of serological studies and electrocardiography were normal.

**Fig. 1:**
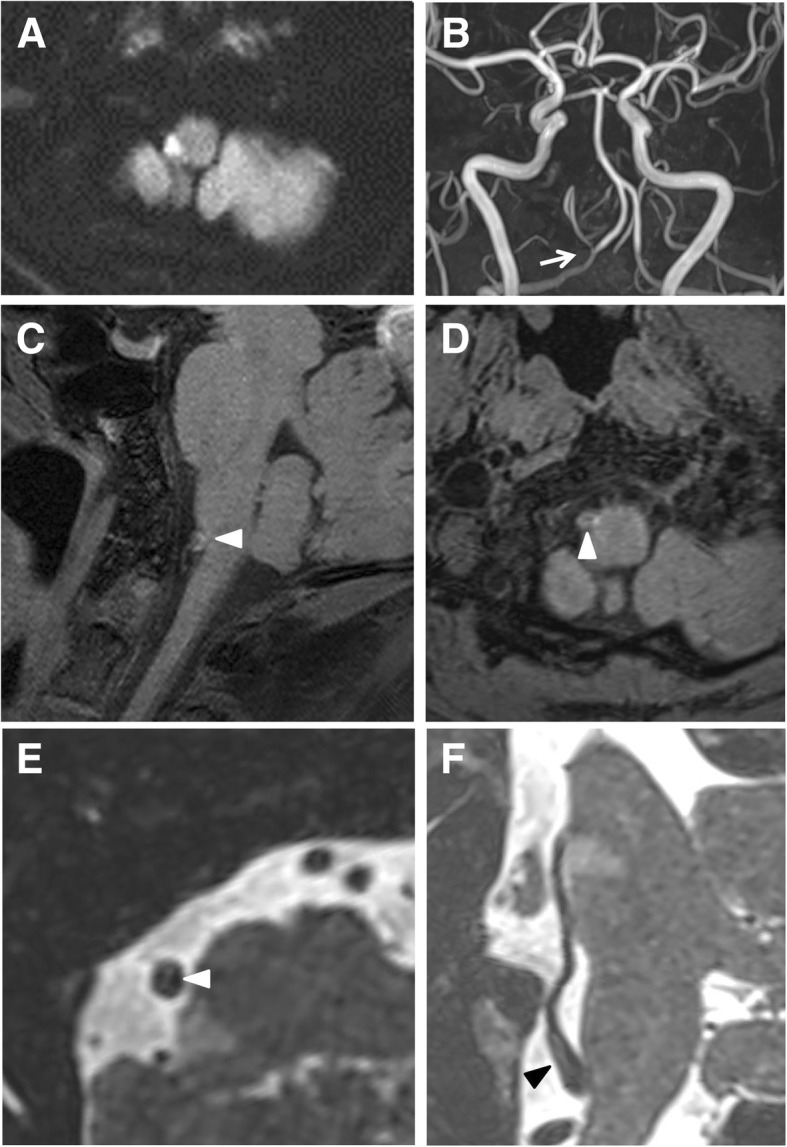
Brain magnetic resonance image (MRI), time-of-flight MR angiography (TOF-MRA), and high-resolution vessel wall MRI (HR vw-MRI) in case 1. Diffusion weighted image (DWI) shows acute infarction in the right lateral medulla (**a**). Stenosis of the right proximal posterior inferior cerebellar artery (PICA) is seen on TOF-MRA (arrow) (**b**). Three-dimensional (3D) sagittal T1 weighted HR vw-MRI reveals a hyperintense intramural hematoma in the right proximal PICA (white arrow head) (**c**). 3D oblique axial T1 weighted HR vw-MRI shows a linear low signal intensity within the right proximal PICA (white arrow head) (**d**). 3D oblique axial T2 weighted HR vw-MRI shows a linear high signal intensity within the right proximal PICA (white arrow head) (**e**). Curved multiplanar reconstruction (MPR) image reconstructed from 3D T2 weighted HR vw-MRI shows an entire intimal flap clearly in one image (black arrowhead) (**f**)

The diagnosis was determined to be an acute ischemic stroke because the diffusion weighted imaging (DWI) of MRI revealed a high signal intensity area in the right lateral medulla. CT angiography (CTA) was taken first at 3 days after the symptom occurred. HR vw-MRI was taken a day after (at the 4th-day). The right PICA was not observed in the first CTA, and partial spontaneous recanalization was observed on the time-of-flight MR angiography (TOF-MRA), which was taken one day after. Moreover, the sagittal 3D T1 weighted HR vw-MRI showed a T1 hyperintense intramural hematoma in the right proximal PICA (Fig. 1). However, a T2 weighted 3D curved MPR image revealed the luminal dilation and intimal flap in the right proximal PICA (Fig. 1). The patient was diagnosed with spontaneous isolated PICA dissection on the basis of the neurological symptoms and imaging. He was treated with antiplatelet agents and the neurological symptoms of the patient improved during course of admission.

### Case 2

A 38-year-old male patient came to the emergency room due to vertigo and vomiting that had started suddenly one day before the visit. The patient complained of a left-sided occipital headache that began two days before the visit. He had no history of trauma within 1 week prior to admission. The patient did not have any underlying diseases, history of migraines, or risk factors for stroke except for smoking and alcohol consumption. His blood pressure was 170/100 mmHg, the pulse rate was 70/min, breathing rate was 20/min, and body temperature was 36.5 °C. A neurological examination showed ataxia in the left upper extremity and the NIH stroke scale was 1. The results of serological studies and electrocardiography were normal.

**Fig. 2:**
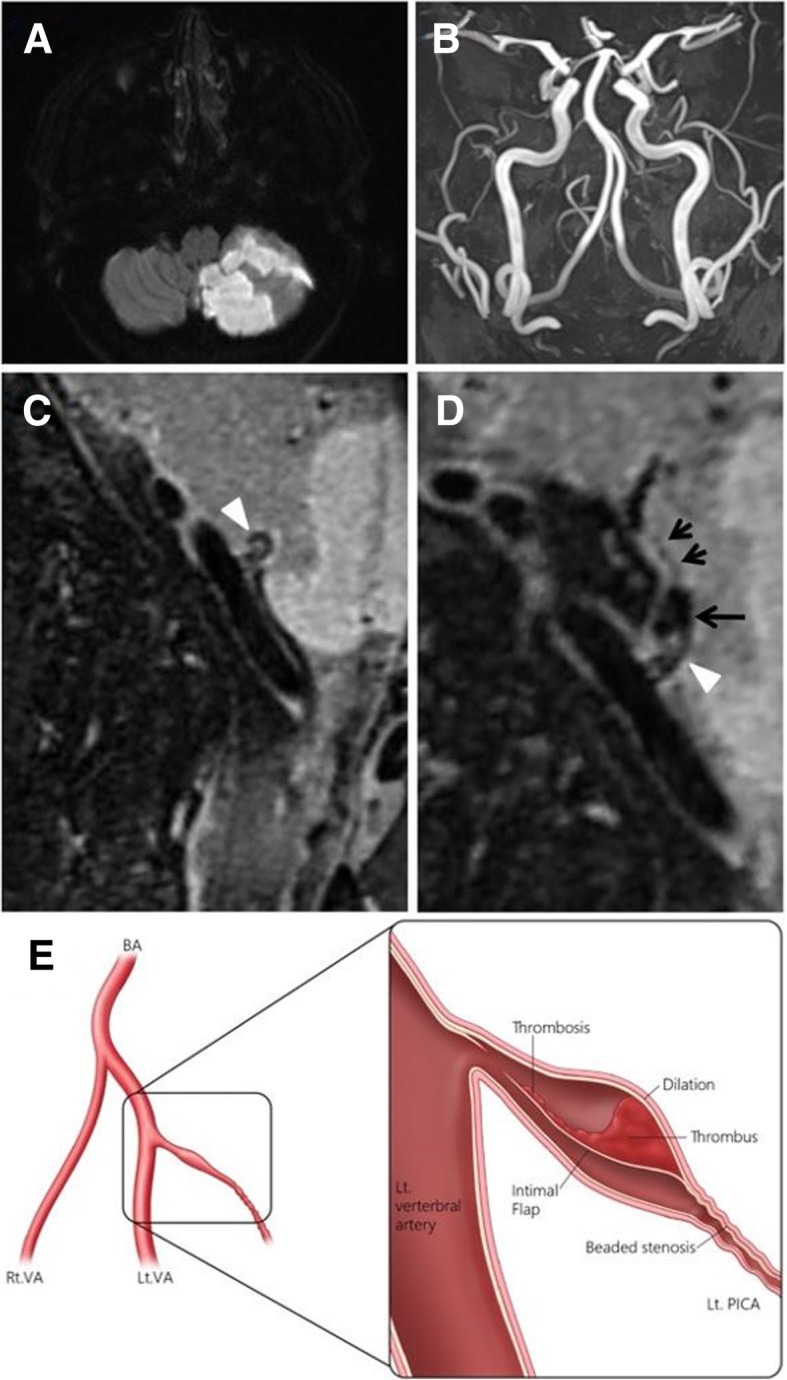
Brain magnetic resonance image (MRI), time-of-flight MR angiography (TOF-MRA), and high-resolution vessel wall MRI (HR vw-MRI) in case 2. Diffusion weighted imaging (DWI) shows acute infarction in the left cerebellar hemisphere (**a**). The left posterior inferior cerebellar artery (PICA) is not seen on TOF-MRA (**b**). Three-dimensional (3D) oblique sagittal T2 weighted HR vw-MRI reveals intraluminal high signal intensity (white arrow head) and mild dilation of the left proximal PICA (**c**). Curved MPR image reconstructed from 3D T2 weighted HR vw-MRI shows an intimal flap (white arrow head) in the left proximal PICA and dilation (long arrow) and beaded stenosis (short arrows) in the left proximal PICA (**d**). Illustration of angiographic images shows an intimal flap and dilation with thrombosis of PICA dissection (**e**). This figure is illustrated by HGK

The patient was determined to have an acute ischemic stroke because the DWI of MRI showed a high signal intensity area in the left PICA territory. CTA was taken at 2 days after the onset of a headache and at 1 day after the onset of neurological symptoms. Moreover, HR vw-MRI was taken at 6 days after the onset of a headache and at 5 days after the onset of neurological symptoms. Although the left PICA was not observed on the TOF-MRA, other vessels including the left vertebral artery were normal. An intraluminal high signal intensity and mild dilation of the left proximal PICA was revealed on routine 3D HR vw-MRI, but pathognomic signs of dissection was not seen. Curved MPR image reconstructed from 3D T2 weighted HR vw-MRI showed dilation and an intimal flap in the left PICA origin and post dilation stenosis in the left proximal PICA (D). (Fig. 2). The patient was diagnosed with spontaneous isolated PICA dissection based on the neurological symptoms and imaging. The patient was treated with antiplatelet agents and discharged after his neurological symptoms improved.

## Discussion and conclusions

In this study, the patients were diagnosed to have spontaneous isolated PICA dissection due to the intimal flap that was observed on the 3D curved MPR images. In particular, the second patient was diagnosed with PICA dissection because an intimal flap was observed on the 3D curved MPR image, even though the routine HR vw-MRI revealed the dilation and stenosis in the left proximal PICA without showing the intramural hematoma.

Chang et al. reported that isolated PICA dissection accounts for 6% of infarctions in the PICA territory [4]. Most cases of isolated PICA dissections were diagnosed by an invasive conventional angiography or an active follow-up study such as MRI [4]. Although isolated PICA dissection is rare, it had been underdiagnosed which leads to the occurrence of ischemic stroke of the posterior circulation [5]. Recent studies have shown that isolated PICA dissection can be diagnosed using HR vw-MRI [1–3]. In most case studies, intramural hematoma, dilated lumen, or pearl-and-string appearance were seen on HR vw-MRI. Although it is hard to consider them as direct signs of PICA dissection, it is very difficult to find the intimal flap or double lumen via an HR vw-MRI because the size of PICA is very small. However, it is possible to obtain further information by the application of 3D curved MPR image to the site suspected to have the dissection because 3D HR vw-MRI can be reconstructed in various directions. The 3D curved MPR image is beneficial for observing tortuous blood vessels such as the PICA because it can visualize the entire vessel course in a single plane. In this report of two cases, 3D curved MPR images were obtained by reconstructing 3D HR vw-MRI, and thus, the intimal flap, which was not seen on the routine images, was found.

T1 hyperintense intramural hematoma is considered to be a pathognomonic finding of arterial dissection. However, it cannot be visualized in all types of dissection because the signal intensity of the hematoma varies by time [6]. A susceptibility-weighted imaging (SWI) study may help the diagnosis of the intramural hematoma; however, the intraluminal thrombosis can be seen as a hypointensity. Therefore, it can be used as an ancillary imaging sequence for diagnosing the dissection because an intramural hematoma is not found in all cases and it should be distinguished from intraluminal thrombosis [3, 7].

Although conventional angiography has been considered to be the key diagnostic tool to diagnose isolated PICA dissection, it has several shortfalls. For example, it does not directly show the vessel wall status, mainly reflects the luminal changes such as pearl-and-string signs, and is difficult to visualize the intimal flap or double lumen directly. Additionally, conventional angiography is invasive and may aggravate the dissection during the procedure [8]. There is no systematic study about CT angiography or cerebral angiography findings for isolated PICA dissection. However, several case studies reported it as occlusion pattern or stenosis [9, 10]. And also, 3D curved MPR imaging consists of reconstructed images and it could be affected by artifact in some extent during the process of the reconstruction.

To conclude, we strongly argue that HR vw-MRI is useful for the early diagnosis of isolated PICA dissection. Furthermore, we firmly believe that the use of 3D curved MPR images could improve the possibility of diagnosing the dissection early because it can confirm the presence of direct signs such as an intimal flap or double lumen.

## Abbreviations

DWI: Diffusion weighted imaging
HR vw-MRI: High-resolution vessel wall magnetic resonance imaging
PICA: Posterior inferior cerebellar artery
SWI: Susceptibility-weighted imaging
TOF-MRA: Time-of-flight MR angiography

## References

[1] Ishitsuka K, Sakaki Y, Sakai S, Uwatoko T, Aibe H, Ago T (2016). Diagnosis and follow-up of posterior inferior cerebellar artery dissection complicated with ischemic stroke assisted by T1-VISTA: a report of two cases. BMC Neurol.

[2] Park HR, Hwang J, Kim YS, Kim J, Jo H, Jung YH (2016). Isolated Posteroinferior cerebellar artery dissection diagnosed by high-resolution Vessel Wall MRI. J Korean Neurol Assoc.

[3] Madokoro Y, Sakurai K, Kato D, Kondo Y, Oomura M, Matsukawa N (2017). Utility of T1-and T2-weighted high-resolution vessel wall imaging for the diagnosis and follow up of isolated posterior inferior cerebellar artery dissection with ischemic stroke: report of 4 cases and review of the literature. J Stroke Cerebrovasc Dis.

[4] Chang F-C, Yong C-S, Huang H-C, Tsai J-Y, Sheng W-Y, Hu H-H (2015). Posterior circulation ischemic stroke caused by arterial dissection: characteristics and predictors of poor outcomes. Cerebrovasc Dis.

[5] Park M-G, Choi J-H, Yang T-I, Oh S-J, Baik SK, Park K-P (2014). Spontaneous isolated posterior inferior cerebellar artery dissection: rare but underdiagnosed cause of ischemic stroke. J Stroke Cerebrovasc Dis.

[6] Habs M, Pfefferkorn T, Cyran CC, Grimm J, Rominger A, Hacker M (2011). Age determination of vessel wall hematoma in spontaneous cervical artery dissection: a multi-sequence 3T cardiovascular magnetic resonance study. J Cardiovasc Magn Reson.

[7] Kim T-W, Choi HS, Koo J, Jung SL, Ahn K-J, Kim B-s (2013). Intramural hematoma detection by susceptibility-weighted imaging in intracranial vertebral artery dissection. Cerebrovasc Dis.

[8] Kaufmann TJ, Huston J, Mandrekar JN, Schleck CD, Thielen KR, Kallmes DF (2007). Complications of diagnostic cerebral angiography: evaluation of 19 826 consecutive patients. Radiology..

[9] Wada Y, Kitano T, Uemura J, Yagita Y. Isolated Posterior Inferior Cerebellar Artery Dissection. Intern Med. 2017 1;56(21):2959–2960. 10.2169/internalmedicine.8780-16 PMC5709650 28924125

[10] Jeong HE, Jang HS, Yu HJ, Roh SY, Choi JW (2014). Spontaneous isolated dissection of the posterior inferior cerebellar artery presenting with lateral medullary infarction. J Neurocrit Care.

